# Community pharmacists' attitudes towards patient leaflets: Exploring perceptions underlying an electronic local production of tailored written information

**DOI:** 10.3934/publichealth.2018.2.189

**Published:** 2018-06-25

**Authors:** Catarina Vitor, Afonso Cavaco

**Affiliations:** Department of Social Pharmacy, Faculty of Pharmacy, University of Lisbon, Lisbon, Portugal

**Keywords:** patient leaflets, tailored information, community pharmacy, qualitative research, Portugal

## Abstract

**Introduction:**

Low health literacy in Portugal, revealed by limited patients' knowledge of their medication, may be improved by written information that is individually tailored for each patient. Tailored content can be produced through computer software and delivered by community pharmacies to patients.

**Objective:**

To assess community pharmacists' real-life management, usage and perceived utility of software developed to produce individually tailored patient leaflets at community pharmacies.

**Methods:**

The software contained five different pharmacist-selected clinical information fields which allows for the adjustment of information to each patient's information needs. Using an exploratory study design, community pharmacists' perceptions were purposively selected and qualitatively assessed. Interviews were recorded, transcribed verbatim and iteratively coded using a thematic approach outlined by attitudinal theory.

**Results:**

Eight participants took part in the study. Emerging codes led to the construction of two main themes: Current PLs usage in Portuguese community pharmacy; and Tailored PLs usage in Portuguese community pharmacy. Pharmacists exhibited a generally positive attitude concerning the relevance and use of patient leaflets to address individual patient's information needs, including an improvement in health literacy. The model was considered effective, functional, satisfying and user-friendly.

**Conclusion:**

Although additional studies are needed, the introduction of a leaflet-tailoring software in Portuguese community pharmacies seems to be feasible as an additional resource to improve the quality of patient information and counselling. The next research steps should address the impact on patients' medicines-related information, including the level to which patients are able to correctly interpret the information and to adjust accordingly their health behaviours.

**Practice implications:**

The software fits present community pharmacy practice and routines, bringing advantages to pharmacists' willingness to deliver meaningful written information to patients, thus contributing to improved patient health literacy.

## 1. Introduction

The Portuguese Health Literacy Survey (HLS-PT) has shown that approximately 50% of the Portuguese population has low health literacy [1], which according to the World Health Organization (WHO) is associated with health issues, such as reduced adherence to medication, poor health choices and inability to manage chronic diseases [2]. Regarding medication adherence, patient-tailored interventions involving counselling from community pharmacists have demonstrated a positive contribution to improved health literacy [3].

When designing and implementing a counselling service, supporting materials for patient education (e.g. printed handouts) are a core resource [4]. These materials require individual tailoring, according to patient's information needs, to impact health outcomes [5]. The offer of personalised health information comprising goals and behaviour options, has shown its value in diverse situations, from increasing physical activity, smoke cessation, incrementing mammography rates, and the improvement of dietary habits and weight loss [6],[7]. Research has shown that patients prefer to receive explanations on how the treatment influence their lifestyle, rather than scientific information on the medicine [8]. The production of individualised printed materials (i.e. adapted written messages tailored to existing patient knowledge and preferences) should be more relevant and useful to the patient, and may contribute to more effective healthcare [9].

Pharmacists play a public health role and should be capable of embracing expanding communications with the public that visit community pharmacies. Pharmaceutical organizations, such as the Royal Pharmaceutical Society, have defended the importance of pharmacists maintaining and developing IT skills, particularly when advising and supporting patients' medicines use [10]. IT tools, such as clinical decision support software, allow pharmacists to influence patient education and empowerment, and enable an increased service quality [11],[12]. These computed-based support tools appear to be more effective when integrated into the routinely used dispensing software, avoiding the potential burden of a separate procedure [13]. Other benefits of additional support tools for pharmacist-patient interactions include increased ability to select appropriate information, improved information sharing and greater patient input [14]. Despite the scarce published evidence, healthcare software programmes supporting tailored interventions in community pharmacies are already a reality [15],[16]. Both pharmacy staff and managers have been shown to believe in the importance of having improved IT capabilities which focus on patient counselling and medication safety [17]. Assuming community pharmacists can provide adequate individualised counselling through computer-printed patient information, the present study aimed to complete the in-house development of a software prototype, designed to produce tailored patient leaflets (PLs) in community pharmacies. It was intended to perform a preliminary evaluation of the usability, content suitability and concept approval of a patient counselling resource in real life context by practising community pharmacists.

## 2. Materials and method

### 2.1. Software prototype development

The desktop software was developed at the Faculty of Pharmacy, University of Lisbon (Portugal), using a MS Access database. The prototype comprised 5 different clinical fields that build the correspondent sections of the printed leaflet. The 5 clinical dimensions include: Condition identifying features (signs and symptoms), acute complications, chronic complications, self-surveillance, and medical surveillance (Table 1). These fields or dimensions were chosen based on existing information, relevance to patient education and patient information preferences described in the literature [8]. Ten different authoritative Portuguese healthcare organizations and their official websites were consulted, with an average of 3 to 4 different sources for each clinical condition. The actual information was uploaded within each field according to the selected conditions, as described in the section.

### 2.2. Software information selection and treatment

The prototype comprised of 9 clinical situations or health conditions with a high prevalence in Portugal: Type 2 Diabetes Mellitus, Cardiovascular disease, Asthma, Chronic pain, Depression, Dyslipidaemia [18], Cancer [19], Sleeping disorders [20], and Flu (as a seasonal clinical condition). In total, there were 34 different information cells (Table 1).

Clinical situation information was collected from website pages and downloadable files which were intended to be accessed by the general population. Existing patient leaflets, either from different pharmaceutical companies or the Portuguese National Association of Pharmacies, were also compiled. The online and printed patient information sources were screened and validated by the research team and one external language expert. The information cells were filled with the compiled information, using plain language rules, applied to written Portuguese [21]. Some information cells were left empty if considered irrelevant to a particular clinical situation. When information was insufficient for a field, it was complemented with other Portuguese healthcare websites, such as *ad-hoc* forums and patients' blogs. These informal sources provided insiders' views and were only used when the authors were clearly identified as valuable experienced patients who regularly posted on their conditions for at least 1 year. This last information comprised, for example, patients' reports on diet (e.g. how to enjoy beneficial food, how to effectively reduced alcohol consumption, etc.) and recommended physical activity (e.g. how to achieve the right frequency). No additional validation work, e.g. using a panel of experts or a consensus group, was completed.

**Table 1.** The 9 clinical situations (CS) and the correspondent 34 leaflet information cells.

Clinical situation	CS-related features	CS acute complications	CS chronic complications	CS Auto-surveillance	CS medical surveillance
Type 2 Diabetes	Characterization	Designation	Designation	What exams should I do?	Exam
Cardiovascular disease	Aetiology	In what consists?	Explanation	Ideal exam conditions	Clinical importance
Asthma	Modifiable factors	Signals and symptoms	Signals and symptoms	Exam special cares	Exam special care
Chronic pain	Unmodifiable factors	Predisposing factors	Predisposing factors	How to interpret results	
Flu	Non-pharmacological therapy	How to react	How to avoid		
Cancer	Pharmacological therapy	How to avoid			
Dyslipidaemia	Eating re-education				
Depression	Beneficial food				
Sleeping disorders	Food to avoid				
	Physical exercise importance				
	Physical exercise frequency				
	Smoking habits				
	Smoking harness				
	Alcohol consumption				
	Everyday details				

Note: CS: Clinical situation.

### 2.3. Software prototype main features

The software interface initially prompts the pharmacist to register the patient name and to select the patient's clinical situation(s). After selecting a clinical situation, all relevant fields are unlocked and can be selected to be printed into the final tailored leaflet. To adequately handle the software, the participating pharmacists were briefly instructed on main operating conditions, including the previous pharmacist-patient exchange to investigate patient's critical information needs.

The prototype allows for history of produced leaflets to be traced-back for a specific patient, including PLs content and production dates. This function is useful to help understand, by following patient interviews, how the information was retained by the patient and if behavioural changes were made.

### 2.4. Exploratory qualitative study: Design and data collection

To assess the software in the real-world (i.e. in the community pharmacy context), an exploratory qualitative study was carried out with practising pharmacists. Participants' inclusion criteria followed a purposive sampling of registered pharmacists, working in urban middle-sized pharmacies, serving up to 300 customers per day and located in the greater Lisbon area. Two pharmacies which were contacted could provide up to 10 pharmacists who were in direct contact with the public and voluntarily accepted participating in the study, after informed consent. Data were mainly obtained by semi-structured individual interviews, supplemented by observation field notes (described below). All interviews took place between December 2015 and January 2016, conducted by the same researcher and audio-taped, with a length of 15 to 20 minutes each. The interview guide was designed to be short, due to time constrains at the practice location. The interviewer questioned participants about: Current usage of patient leaflets in daily work, their attitudes in relation to the possible use of computerized production of tailored PLs within their professional routines, and after the prototype demonstration and actual use, their perceptions on the feasibility and utility of the proposed software. Software usability questions were adapted from the System Usability Scale [22]. During the interview, participants were also subject to direct observation of the software usage, through an unstructured approach, with the observer noting how participants handled the software interface and registering any non-verbal reactions.

The interview schedule was initially assessed by 2 independent academic experts and then submitted to a pre-test with an experienced practitioner, prior to the actual field work. No administration issues were identified, and no changes deemed necessary to reach the study objectives.

### 2.5. Data analysis

The transcribed verbatim interviews and the few observation notes were imported into the software QSR^®^ NVIVO 11 Pro. This software was used to organize and retrieve all information, following a thematic analysis inspired by attitudinal theory [23]. The analytical framework was based on cognitive, affective and behavioural participants' accounts. The final coding emerged through a reflective procedure and systematic codes comparison. The codes and themes were found independently from the questioning sequence, with different codes emerging naturally from the open coding of the raw data, thus assuring a free search into the participants' attitudes. Relevant participants quotations were translated from Portuguese to English, preserving as much as possible the core idea, although minor adjustments were made to ensure legible written English. Data were initially analysed by two independent researchers until a stable coding frame (at the 3rd interview), and then continued by a single coder, who coded data subject to consensus validation.

## 3. Results

The present study results were obtained from a total of 6 female and 2 male participants, reflecting the gender divide working at community level. A sample mean age of 39 years old (range 27–55) was seen along with an average work experience of 14 years (range 2.5–30). Data saturation was reached on the 6^th^ interview as no new data, themes or coding emerged. Total redundancy was confirmed at the 8^th^ interview.

The direct observation of participants handling the software did not uncovered any systematic difficulty or any unexpected non-verbal reaction. Regarding the transcribed interview data, two main themes were developed (Table 2):

- Theme A—current PLs usage in Portuguese community pharmacy;
- Theme B—tailored PLs usage in Portuguese community pharmacy.

Theme A comprised 5 categories: Written information, PLs subject-matter, PLs content quality, PLs quality screening and PLs distribution. In turn, theme B comprised 6 categories: Tailored written information, prototype usability assessment, software usage, software update and content insert, willingness to pay and software evaluation. To illustrate themes and categories, translated participant quotations are given (*Qi*), as well as participant identification by their initials (e.g. *CL*), gender (e.g. *female*) and age.

**Table 2.** Coding tree.

Theme	Sub-themes	Categories
Theme A—Current PLs usage in PT CP	Theme A.1—PLs cognitive-related expressions	A.1.1—Written information
		A.1.2—PLs subject-matter
		A.1.3—PLs content quality
	Theme A.2—PLs affective & behaviour-related expressions	A.2.1—PLs quality screening
		A.2.2—PLs distribution
Theme B—Tailored PLs local production in PT CP	Theme B.1—Tailored PLs cognitive-related expressions	B.1.1—Tailored written information
		B.1.2—Prototype usability assessment
	Theme B.2—Tailored PLs behaviour-related expressions	B.2.1—Software usage
		B.2.2—Software update and content insert
		B.2.3—Willingness to pay
	Theme B.3—Tailored PLs affective-related expressions	B.3.1—Software evaluation

Note: CP: Community pharmacy; PLs: Patient leaflets; PT: Portuguese.

### 3.1. Theme A—current PLs usage in community pharmacy

#### 3.1.1. Theme A.1—PLs cognitive-related expressions

##### A.1.1—Written information

In general, participants considered written information availability as advantageous for patients, with emphasis on the expected outcomes, such as increased patient knowledge, medication adherence, patient health protection and the possibility of reading the materials later. *Q1 “Patients can manage their therapeutics with a higher accuracy” TG, f, 55 yrs.*

On a broader level, a participant identified written health information accessibility as a possible contributing factor to the reinforcement of community health. *Q2 “If that information is available, we all win” CL, f, 38 yrs.*

Despite considering PLs informative and advantageous, participants believed distribution to be difficult due to lack of time during the interaction with patients. Hence, the actual pharmacy delivery of health and medicines written information was, overall, reviewed negatively. For example, participants thought the software and resulting leaflets could be a possible waste of time, susceptible of misinterpretation by the patients and maybe financially disadvantageous. *Q3 “In terms of time, it may not be financially profitable” VS, m, 28 yrs.*

##### A.1.2—PLs subject-matter

Regarding content, participants offered expressions on industry- and institutionally-developed PLs. On one hand, participants agreed that industry PLs mainly have the marketing goal of promoting a specific brand or product, instead of providing independent and evidence-based information about a health condition and/or its treatment. One clear area of such market-oriented PLs is the one addressing minor ailments and over-the-counter medicines. *Q4 “[market-oriented] PLs contain information about the range of products they sell” SM, f, 51 yrs*.

On the other hand, whenever PLs were considered informative and relevant for a clinical situation, their source was pointed as institutional, usually by the Portuguese National Pharmacies Association (ANF), or locally-developed and electronically produced by Sifarma^®^ (ANF's managing software used by most Portuguese pharmacies). *Q5 “Distributed by ANF, they [PLs] relate more to a pathology, (e.g.) diabetes, hypertension, asthma” TG, f, 55 yrs.*

##### A.1.3—PLs content quality

Participants gave mixed opinions concerning the PLs content quality, mainly based on their source. Positive views were obtained concerning the clinically-oriented PLs, evaluated as presenting good quality, and being informative and clear (i.e. being adequate for the clinical situation they are addressing). Regarding the industry-developed PLs, opinions were generally positive when the provided information was sufficient to generate a purchase of the publicised product. *Q6 “Some are effectively clear, explain and generate sales” IP, f, 27 yrs.*

In contrast, negative views consisted of perceptions of inefficiency to inform patients and a possible random distribution of PLs without considering individual information needs. In addition, a specific negative review of the industry PLs was the variable quality in information content and accuracy. *Q7 “The quality of industry-defined patient leaflets is more variable” TG, f, 55 yrs.* This was also addressed in an affective and behavioural manner, presented in the section A.2.1—PLs quality screening.

#### 3.1.2. Theme A.2—PLs affective & behaviour-related expressions

##### A.2.1—PLs quality screening

From participants' accounts emerged an affective and behavioural-based code regarding PLs quality. Since PLs raise suspicions about their overall quality, one participant stated that PLs were screened and assessed prior to patient delivery. *Q8 “We analyse the patient leaflets we deliver” CL, f, 38 yrs.*

##### A.2.2—PLs distribution

PLs distribution was presented as highly dependable on PLs availability at the pharmacy, since ANF did not always provide the needed amount of PLs, and it was not possible to print them locally with the same final quality. Hence, participants considered their production and distribution unfeasible, while expressing concerns of surpassing the usual pharmacy visit duration when handling PLs to patients, beyond what might be acceptable to them. One participant offered the alternative of delivering the produced PLs later, to shorten the time gap. *Q9 “The service time must not be long, it [PL] would have to be delivered later” TG, f, 55 yrs.* Regardless of the availability, participants recognised that PLs should be distributed according to the information needs of the patient, instead of wasting an information resource, by randomly delivering the available PLs to patients. *Q10 “I can give a PL without utility, if I do not always select who receives it” CL, f, 38 yrs.*

### 3.2. Theme B—Tailored PLs local production in community pharmacy

#### 3.2.1. Theme B.1—Tailored PLs cognitive-related expressions

##### B.1.1—Tailored written information

The interview schedule contained broad-spectrum questions about the experienced prototype to produce tailored PLs. During the interviews, all participants were cognitively clear about the advantages of individualised patient information, in terms of information suitability, satisfaction and benefits.

##### B.1.2—Prototype usability assessment

After receiving a demonstration and experimenting the software, participants were encouraged to comment as users on their satisfaction or dissatisfaction with the software features, including the overall format and layout, features that worked or did not work well, and whether they believed the prototype could enhance PLs distribution. Participants were also encouraged to recommend content additions or layout changes.

Overall, contributions were based on software features, such as interface organization *Q11 “Put it [conditions, etc.] in alphabetical order, for the selection to be quicker” CL, f, 38 yrs*; effectiveness *Q12 “It might save time in the following pharmacists' interventions” CL, f, 38 yrs;* and function *Q13 “[It needs] integration with Sifarma^®^. A parallel application falls into disuse because the pharmacy is always full, and we need to serve customers as soon as possible, thus there is less time to use a software outside Sifarma^®^” PV, m, 33 yrs.* Furthermore, the software was considered easy to use for someone with basic knowledge of informatics.

Concerning integration with the work flow, some positive opinions were also expressed, such as the easiness of software access and leaflet production, differentiation of customer service, and suitability of this information-complementing strategy. *Q14 “It can be used to consolidate oral counselling” SM, f, 51 yrs.* Negative opinions were again focused on the time consumed to produce the tailored PLs and the need for Sifarma^®^ integration. *Q15 “The fact that is tailored takes some time, to select the information” VS, m, 28 yrs.*

#### 3.2.2. Theme B.2—Tailored PLs behaviour-related expressions

##### Theme B.2.1—Software usage

Expressions on full usage of the software in day-to-day work routine in community pharmacy were also collected. Approximately half of the participants accepted full usage of the software based on the prototype shown because of its simple and ready-to-use concept. *Q16 “Yes, easiness of introduction” MG, f, 37 yrs.*

However, when asked about regular usage, participants provided a multitude of answers. Positive views considered regular usage possible because it would increase service quality and patient information, assuming the possible integration and usage in daily work routine. Nevertheless, motivation of the pharmacy staff was pointed out as a critical factor. *Q17 “Regularly yes, on this pharmacy, because we have a work team that likes to differentiate itself, do more and better” PV, m, 33 yrs.* Negative views once again strongly highlighted lack of time and impossibility of extending service time. In the light of Q13, an effective software implementation, e.g. a possible integration in the Sifarma^®^ software, could increase its regular usage.

##### Theme B.2.2—Software update and content insert

Participants outlined that software updates should be conducted centrally, in a standardised update process. This was considered advantageous because if a patient would collect two PLs in different pharmacies, the probability of receiving different information and getting confused would decrease. Although being relevant, this idea conveys a sense of less direct accountability for the quality of the information content, as well as less opportunities to conduct detailed changes for an enhanced leaflet tailoring. One participant mentioned the assigned team for the updates should additionally collect software users' thoughts and ideas. *Q18 “It should receive pharmacies opinions” PV, m, 33 yrs.*

Concerning additional information fields, participants expressed that the software should allow users to directly add information that was not comprised in the computerized leaflet production. *Q19 “It must allow a field where it is possible to occasionally add information” TG, f, 55 yrs.*

##### Theme B.2.3—Willingness to pay

Innovation is always associated with financial costs. Participants pointed out that software acquisition would be important, but it was not seen as an indispensable pharmacy equipment. Thus, participants' willingness to pay depended generally on the software price. *Q20 “Depends on the price, because it valorises a little bit the pharmacy [service]” VS, m, 28 yrs.* Furthermore, willingness to pay was also related to participants' beliefs on the software added-benefits. The benefits pointed out were patient valorisation *Q21 “Because it valorises the patient” CL, f, 38 yrs*; disease prevention and valorisation of the pharmacy service *Q22 “Because is an asset to pharmacy service” CL, f, 38 yrs.*

#### 3.2.3. Theme B.3—Tailored PLs affective-related expressions

##### Theme B.3.1—Software evaluation

As the 34 information cells are not always completely independent from each other, participants believed that cells or section number could be reduced to decrease the probability of repeated/similar information in the printed leaflet.

Nevertheless, overall comments regarding concept approval were very positive and undoubtedly revealed the software acceptance. *Q23 “By tailoring information for the patient, it [the software] might be able to deliver better information” MG, f, 37 yrs*.

## 4. Discussion

Pharmacists' counselling enables professional satisfaction and fulfilment, and improve patients' perception of pharmacies as a “caring” service [24]. Written instructions help patients avoid mistakes caused by poor recall of the healthcare professional's recommendations [25]. Moreover, patients point out personalised counselling in community pharmacy as a resource capable to improve their health literacy, especially in relation to their overall understanding of medicines [26].

All participants were cognitively clear about the advantages of individualised patient information, in terms of information suitability, satisfaction and benefits. These findings are in accordance with previous studies [6],[9],[27]. Participants demonstrated interest in using a computer software to facilitate the automatic local production of individualised PLs. According to Kreuter *et al*., pharmacists' close proximity to the community furthers their knowledge of the recipient. This increases the level of communication tailoring and production of information materials to address the patient's specific needs [28]. However, only limited evidence of software programmes producing tailored interventions in pharmacies exists [15],[16].

Although interviewed pharmacists considered PLs important patient information tools, they did not find their distribution feasible in practice, which suggests two professional issues: Firstly, a degree of desirability bias, maybe justified by the link of the interviewer to the authors (i.e., university peers); secondly, the inexistence of a structured patient counselling, where time spent with the patient beyond normal selling time is not recognised to be part of the community pharmacy healthcare mission and a daily task. Moreover, postponing PLs delivery as suggested also does not allow pharmacists to explain the content of the PLs to patients, as required by the best counselling practices.

The perceptions offered on who should receive PLs demonstrated the belief that only patients with information needs should be offered a PL. This follows previous findings by Abrams *et al*. whose study highlighted the importance of understanding who should receive tailored messages and the extent of their impact [29]. Another important finding was that training was considered necessary for pharmacists to be able to produce the best tailored PLs possible, also described in the published literature [30].

Participants in favour of the prototype showed an overall positive attitude, considering it valuable and an added-benefit to the pharmacy service, similarly to what was seen in other studies [11],[12]. Willingness to pay for such an application was found to be closely linked to the belief that the software could improve patient counselling. Typically, interventions that are relatively brief, less costly and less intensive can be very broadly disseminated [29] making use of the current wide spread of computers. Nevertheless, overall satisfaction with the proposed prototype reveals that participants believe in the use of technological solutions to improve patient counselling [17].

Also, improvements based on the suggestions already gathered could be made to enhance the initial prototype for pharmacy practice, particularly the integration in the Sifarma^®^ software [13]. An alternative delivery system, such as a pdf sent-out by e-mail, in which PLs content would be explained orally or using a technological resource during the pharmacy visit, thus saving printing time, could be considered in future studies. Furthermore, it would be interesting to assess what information cells were considered to be possibly superfluous or lacking in the software database. Ethical questions such as patient data protection, storage and sharing should be considered as well [31].

Although not directly addressed by participants, the software history function may help in answering questions related to developing patient education interventions, such as “are tailored messages more effective with some health behaviours and/or some individuals more than others?” and “what types of tailored print materials have been the most effective?” [32].

Patients' perceptions should be collected to shed light on the software tailoring suitability and tailored PLs usefulness [33], ideally with a longer-term evaluation of outcomes (e.g. the impact on improving patient health literacy and health behaviours) [28]. And, if possible, it could be helpful to link with other community pharmacy services to demonstrate benefits in public health, such as therapeutic adherence [34] and healthcare savings [35].

Finally, these qualitative findings were considered transferable to most Portuguese community pharmacies, since the chosen participants exemplify the active pharmacy workforce and all pharmacies have computers available, which allows for new software integration.

### 4.1. Study limitations

Further studies are required to understand the extent and criteria used on the professional screening and validation of existing patient leaflets in community pharmacies, being desirable a deeper qualitative assessment. This data should confirm the actual concept approval, by ensuring PLs apparent acceptance does not reflect a professional desirability bias.

To improve the credibility and transferability of these preliminary qualitative findings, future studies should focus on other sample types, based on diverse pharmacy practice locations, so that results on software acceptability and utility can better serve the specificities of the different Portuguese regions.

Finally, there was no research on PLs language, formatting, and contextual factors that seems to be intuitively important to the effective diffusion of tailored communications [29]. Regulations advise that patient information should be provided “in consumer-understandable language” [25]. Although the present study was not able to assess patients' actual use and impact of the leaflets produced, the information database as well as leaflets layout were constructed under the orientation of the Federal Plain Language Guidelines [21].

## 5. Conclusions

This work showed the potential relevance of a novel system to locally produce tailored patient leaflets. The present prototype fits the current smart, logical and cutting-edge trend of implementing eHealth products and services. Portuguese community pharmacies are well equipped and using some eHealth resources, but not necessarily those resources centred in patients' individual needs. The proposed prototype was positively praised by practitioners towards this goal, which suggests the potential of this software to be included into daily community pharmacy work, although further studies are needed to fully assess the prototype feasibility and content suitability. Nevertheless, tailoring patient leaflets to an optimal content and layout is a promising development avenue.
